# Supplementary material for: Genomic Characterization of Escherichia coli Isolates from Alpaca Crias (Vicugna pacos) in the Peruvian Highlands: Insights into Functional Diversity and Pathogenicity
Source: Microorganisms. 2025 Jun 30;13(7):1533. doi: 10.3390/microorganisms13071533 (PMC12298738; doi:10.3390/microorganisms13071533)
Supplement: Supplementary file 1 [file microorganisms-13-01533-s001.zip › supplementary_Figures.pdf]

a)

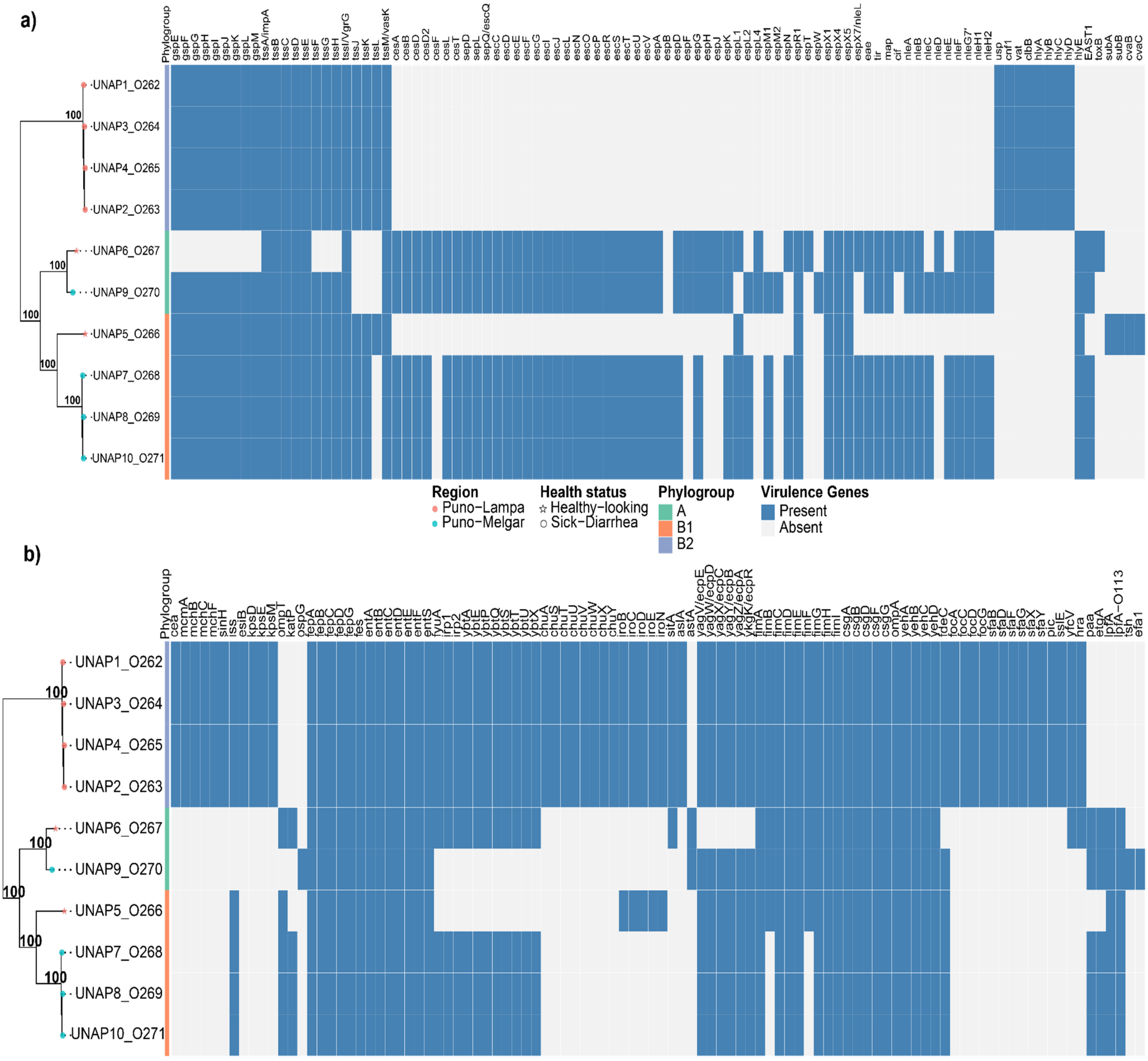

Figure S1. Binary heatmap showing the presence/absence of all identified virulence factors across the 10 *E. coli* isolates. A phylogenomic tree of these strains, annotated with phylogroup, MLST, serotype, and health status, is displayed alongside it. Tree tips are marked with stars for isolates from healthy alpacas and with circles for DEC isolates. (a) First part of the heatmap, (b) Second part of the heatmap.

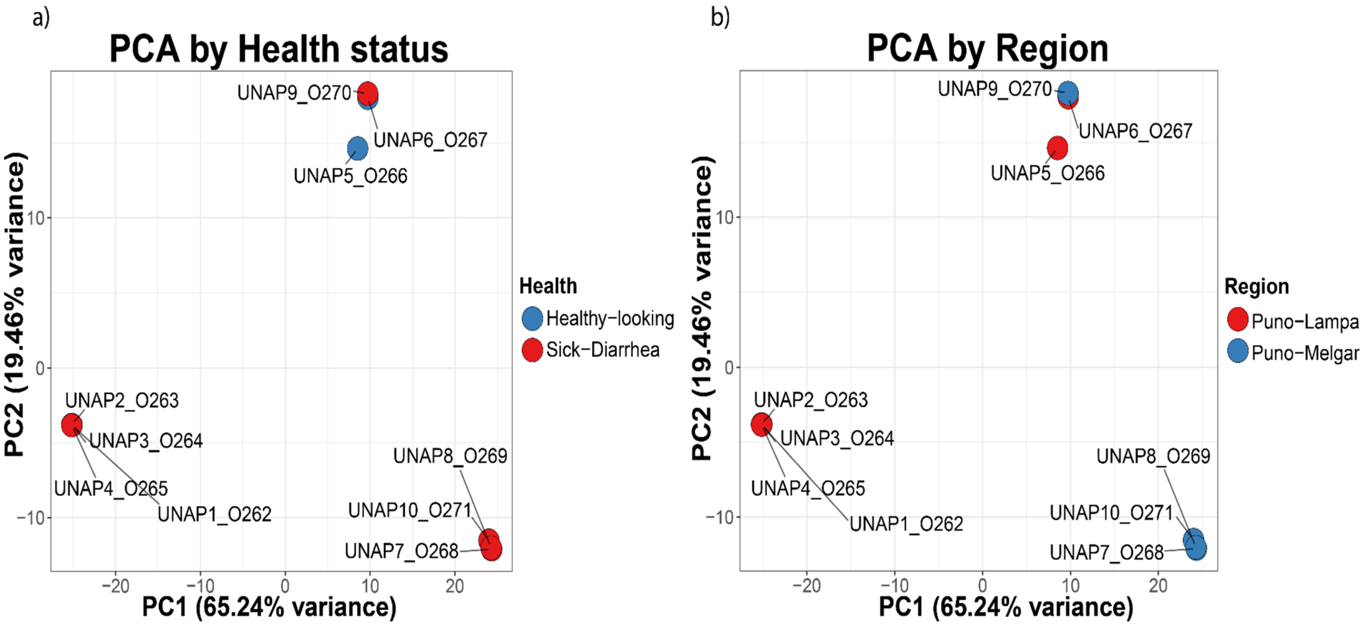

Figure S2. Principal Component Analysis (PCA) of the E. coli accessory genome composition. Each point represents a genome, clustered based on the presence-absence of accessory genes. Colors indicate (a) phylogroup classification and (b) MLST. The clustering of isolates was primarily driven by variation in accessory genome content rather than phylogeny or MLST.

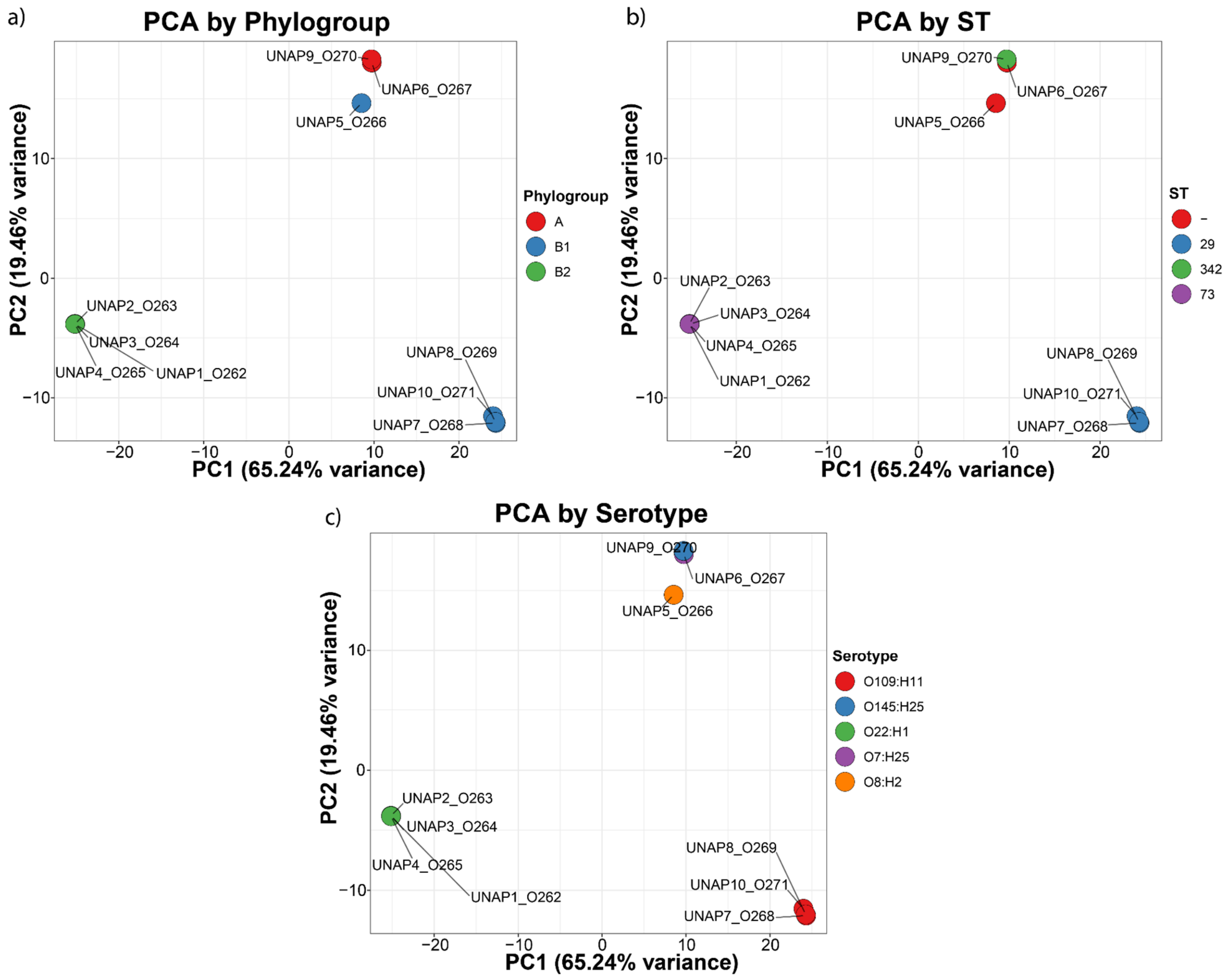

Figure S3. Principal Component Analysis (PCA) of the accessory genome composition of the ten *E. coli* isolated from alpacas for this study. Each point represents a genome, clustered based on the presence-absence of accessory genes. Colors indicate (a) phylogroup classification,(b) MLST and (c) Serotype.

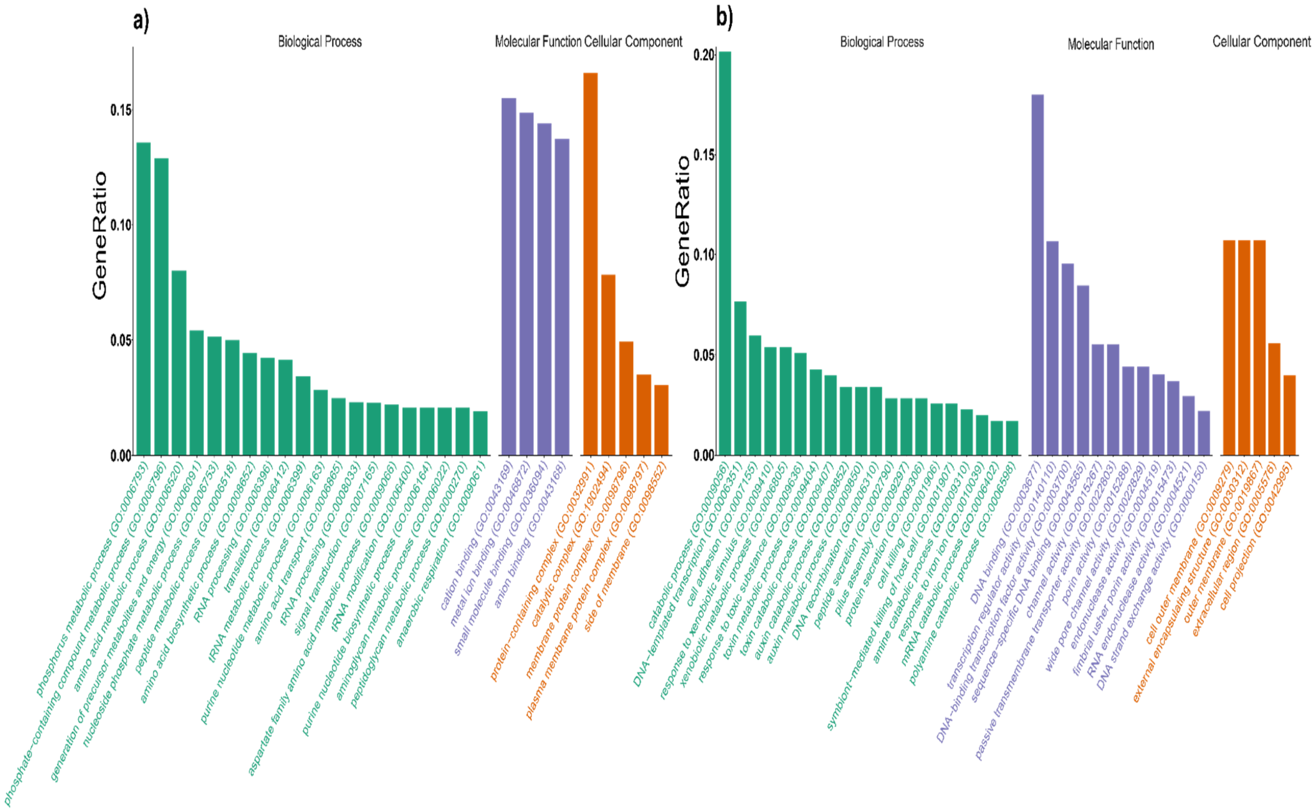

Figure S4: GO enrichment analysis based on the Pangenome of *E. coli* genomes from Alpacas of the present study (10). (a) Core genome (b) Accessory genome.

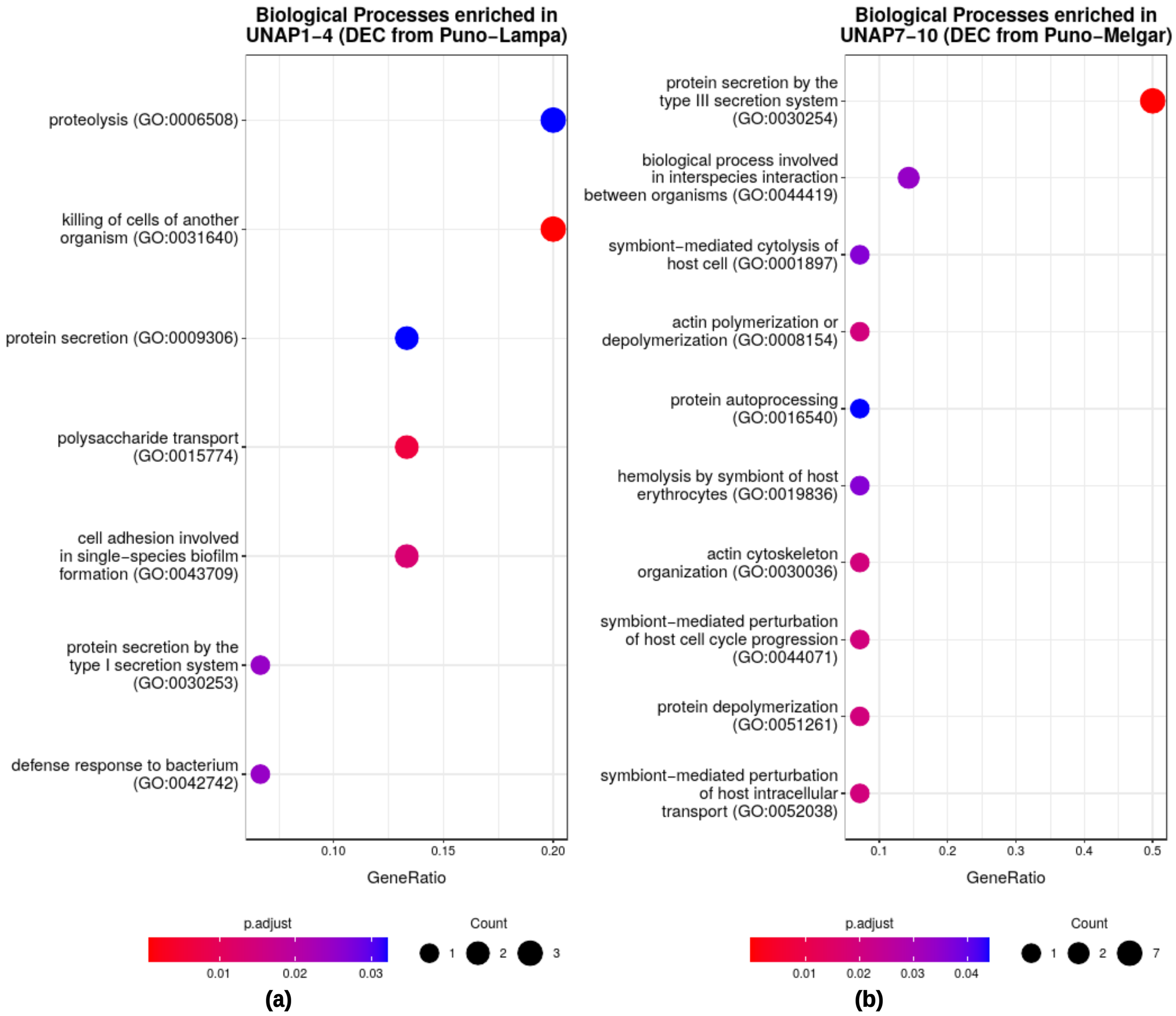

Figure S5: GO enrichment analysis based on the virulence genes in both groups of DEC E- coli from the two sampling regions. Biological processes enriched in (a) UNAP1-4, DEC strains from Puno-Lampa. (b) UNAP7-10, DEC and EPEC strains from Puno-Melgar.

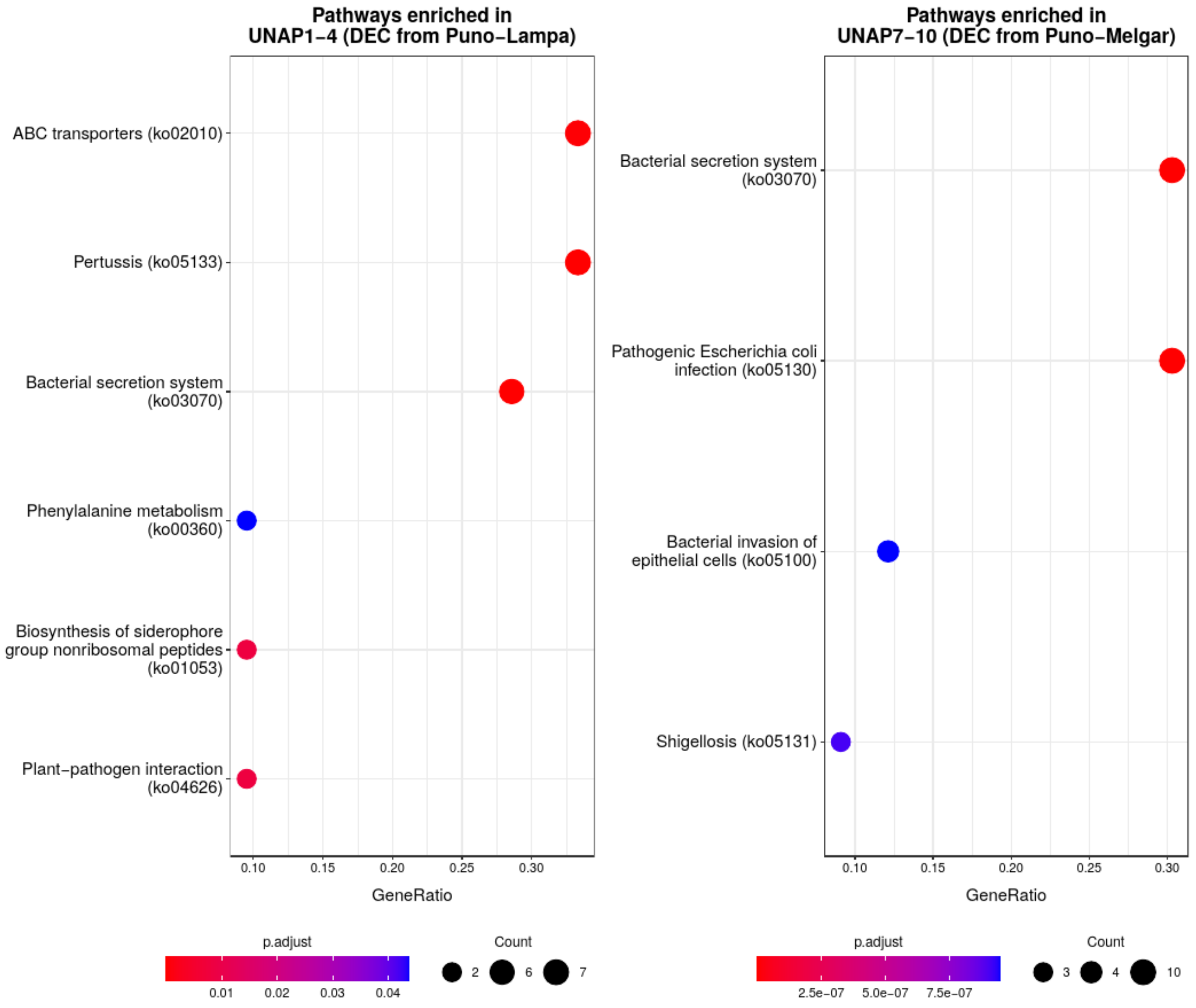

Figure S6: KEGG Pathway enrichment analysis based on the unique virulence genes found in the groups of DEC *E. coli* from the two sampling regions. Pathways enriched in (a) UNAP1-4, DEC strains from Puno-Lampa. (b) UNAP7-10, DEC and EPEC strains from Puno-Melgar.

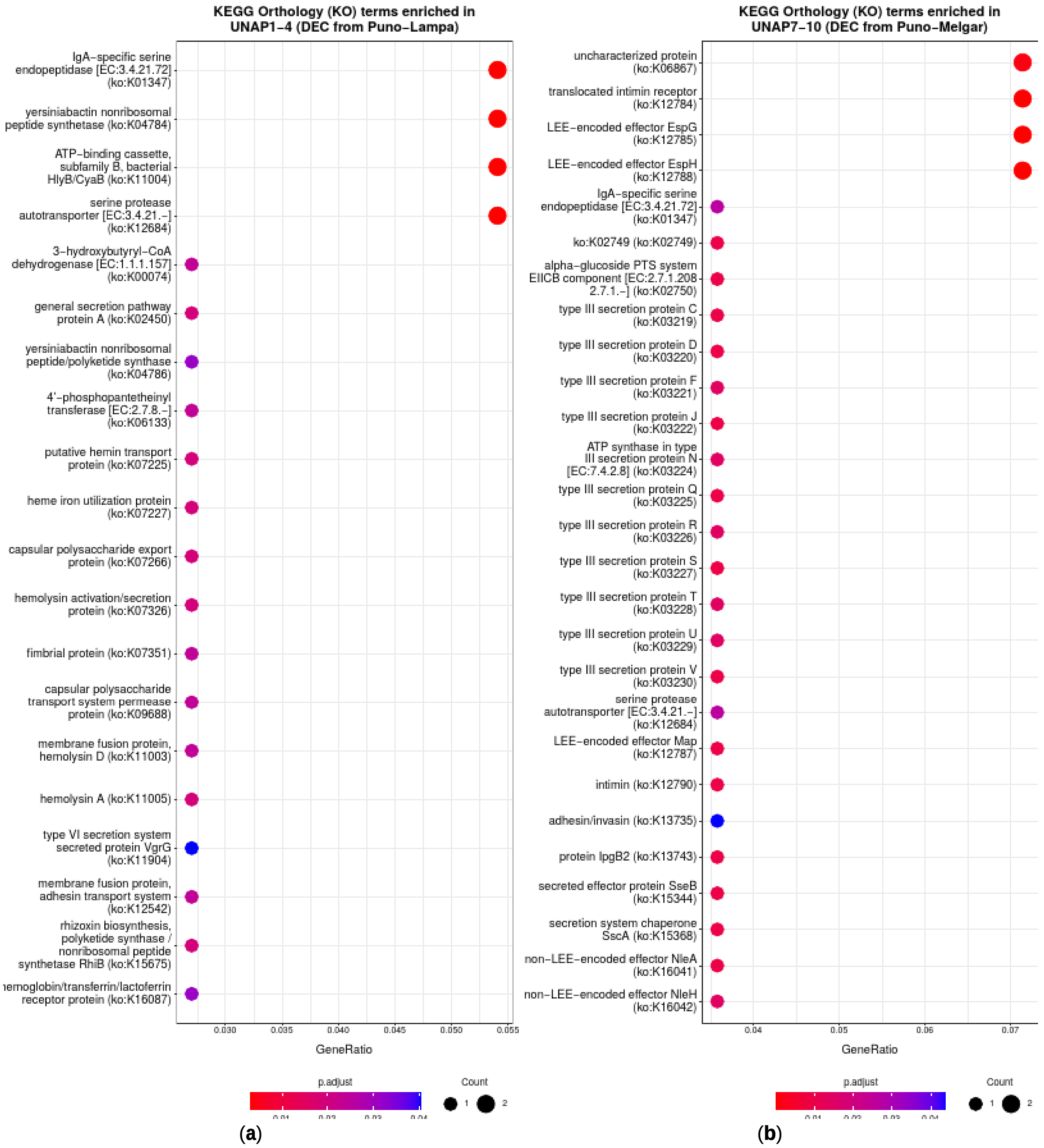

Figure S7: KEGG Orthology (KO) enrichment analysis based on the unique virulence genes found in the groups of DEC *E. coli* from the two sampling regions. KO terms enriched in (a) UNAP1-4, DEC strains from Puno-Lampa. (b) UNAP7-10, DEC and EPEC strains from Puno-Melgar.

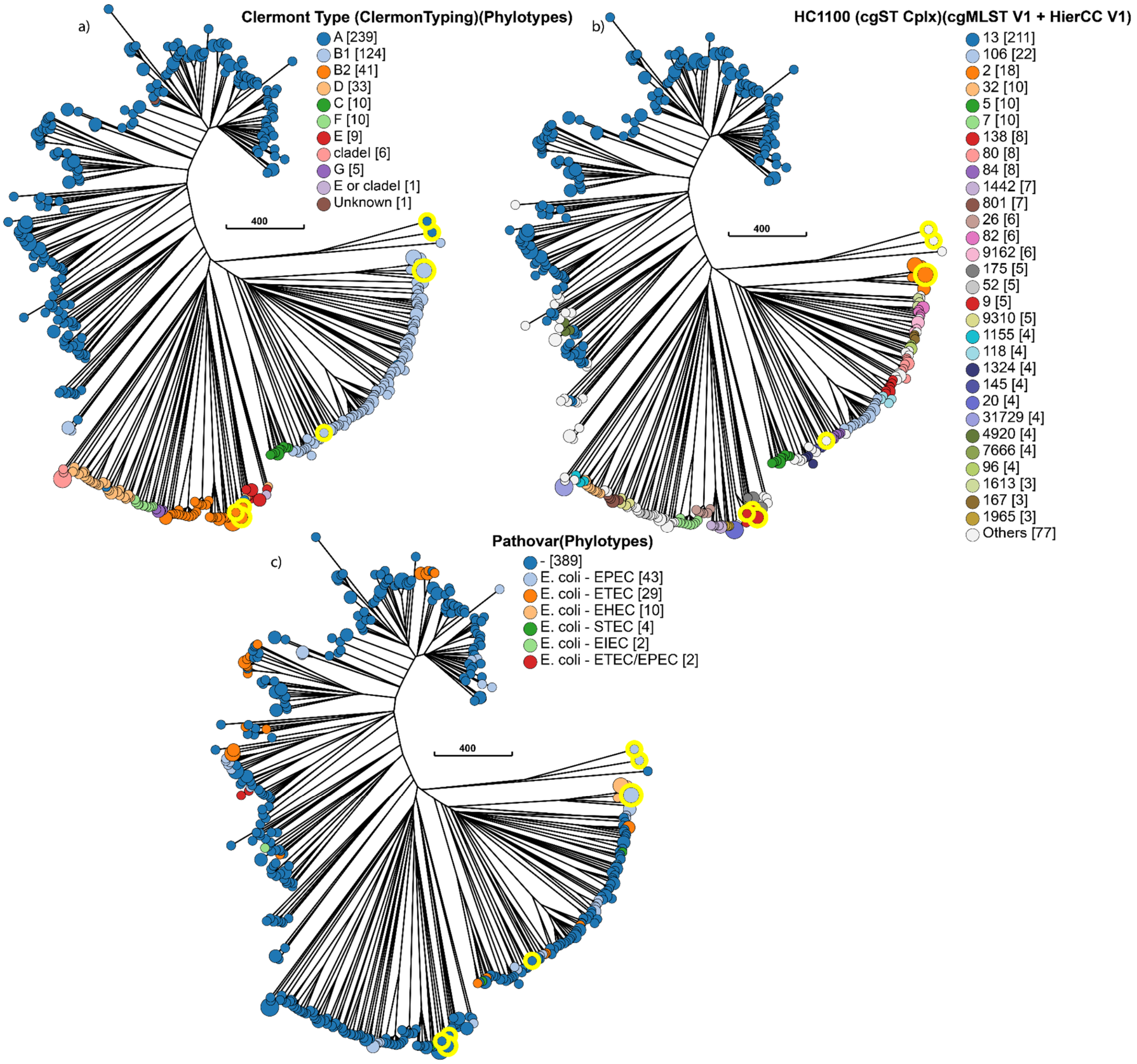

Figure S8. Minimum Spanning Trees (MST) based on the cgMLST V1 + Hierarchical Clustering (HierCC V1) scheme, including 479 *Escherichia coli* genomes from Peru available in EnteroBase and the 10 *E. coli* isolated from alpacas for this study. The tree was constructed using the Neighbor-joining (RapidNJ) algorithm and visualized with GrapeTree in EnteroBase. Nodes are colored according to (a) Phylogroups; (b) HC1100 (CgST Cplx) and (c) Pathovar. The red dots highlighted in yellow are the *E. coli* genomes from Alpacas of the present study (10). The numbers in brackets are the number of isolates belonging to a phylogroup (a), pathovar (b) or cgST Cplx (c).
